# Specialist alcohol inpatient treatment admissions and non-specialist hospital admissions for alcohol withdrawal in England: an inverse relationship

**DOI:** 10.1093/alcalc/agaa086

**Published:** 2020-09-04

**Authors:** Thomas Phillips, Chao Huang, Emmert Roberts, Colin Drummond

**Affiliations:** Institute for Clinical and Applied Health Research, Allam Medical Building, University of Hull, Cottingham Road, Hull HU6 7RX, UK; National Addiction Centre, Institute of Psychiatry, Psychology and Neuroscience (IoPPN), King's College London, 4 Windsor Walk, London SE5 8AF, UK; Hull York Medical School, Allam Medical Building, University of Hull, Cottingham Road, Hull HU6 7RX, UK; National Addiction Centre, Institute of Psychiatry, Psychology and Neuroscience (IoPPN), King's College London, 4 Windsor Walk, London SE5 8AF, UK; South London and the Maudsley NHS Foundation Trust, Bethlem Royal Hospital, Monks Orchard Road, Beckenham BR3 3BX, UK; National Addiction Centre, Institute of Psychiatry, Psychology and Neuroscience (IoPPN), King's College London, 4 Windsor Walk, London SE5 8AF, UK; South London and the Maudsley NHS Foundation Trust, Bethlem Royal Hospital, Monks Orchard Road, Beckenham BR3 3BX, UK

## Abstract

**Aims:**

We assessed the relationship between specialist and non-specialist admissions for alcohol withdrawal since the introduction of the UK government Health and Social Care Act in 2012.

**Methods:**

Using publicly available national data sets from 2009 to 2019, we compared the number of alcohol withdrawal admissions and estimated costs in specialist and non-specialist treatment settings.

**Results:**

A significant negative correlation providing strong evidence of an association was observed between the fall in specialist and rise in non-specialist admissions. Significant cost reductions within specialist services were displaced to non-specialist settings.

**Conclusions:**

The shift in demand from specialist to non-specialist alcohol admissions due to policy changes in England should be reversed by specialist workforce investment to improve outcomes. In the meantime, non-specialist services and staff must be resourced and equipped to meet the complex needs of these service users.

## BACKGROUND

In England, alcohol-related hospital admissions have risen from 493,760 in 2003/04 to over 1.26m in 2018/19 (Public Health England, 2020). A recent study estimated 1 in 5 patients admitted to hospital experience harmful drinking, with 1 in 10 experiencing alcohol dependence (Roberts *et al*., 2019). A disproportionate impact of alcohol on the National Health Service (NHS) is exerted by those with chronic alcohol disorders accessing care via emergency departments (Phillips *et al*., 2019). Alcohol-related disorders have been estimated to cost the NHS £3.5bn per year (Department of Health, 2013).

Effective treatment services should respond to the full spectrum of risks and acute, chronic and complex needs (Babor *et al*., 2008), with inpatient care treating those at greatest risk of severe presentations by providing medically assisted alcohol withdrawal (National Institute for Health and Care Excellence (NICE), 2010, 2011). However, reductions in public health funding in England to commission specialist alcohol treatment since the introduction of the Health and Social Care Act (2012) (HSCA) have been associated with the closure of numerous specialist inpatient units and increasing pressures on acute hospital services (Robertson *et al*., 2017; HC Deb 2019; Drummond, 2017).

We examined publicly available data to estimate the potential impact of the HSCA policy changes before and after 2013 in relation to access to specialist and non-specialist inpatient admissions for alcohol withdrawal care.

## METHODS AND DESIGN

We conducted secondary analysis of publicly available official national data from 2009 to 2019 to examine the associations between specialist and non-specialist admissions following the introduction of the HSCA.

All adult services commissioned to provide specialist alcohol treatment in England report the number of inpatient treatment episodes to the National Drug Treatment Monitoring System (NDTMS). From 2009/10 to 2012/13 data are available on the number of service users accessing ‘planned inpatient alcohol detoxification’ to manage alcohol withdrawal. This definition was changed in 2013/14 to identify those service users accessing ‘inpatient prescribing interventions’. We have treated data on ‘inpatient alcohol detoxification’ and ‘inpatient prescribing interventions’ with equivalence and defined these as ‘specialist admissions’.

Hospital Episode Statistics (HES) are provided by NHS Digital, and include the annual number of non-specialist hospital admissions for a primary or secondary diagnosis of alcohol withdrawal (Public Health England, 2020). The number of admissions for alcohol withdrawal is based on methodology originally developed by the North West Public Health Observatory (Jones *et al*., 2008), which avoids double counting of specific alcohol diagnoses. We used diagnostic codes defined by the International Classification of Diseases, tenth version (ICD-10; WHO, 1992), to identify the number of alcohol withdrawal admissions (F10.3) from 2009 to 2019, which we have defined as ‘non-specialist admissions’. Admissions recorded as ‘withdrawal state with delirium’ (F10.4) were excluded from this analysis as these conditions represent a medical emergency requiring acute hospital settings only.

Our study also examined the cost of admissions in the two settings. The estimated daily cost for specialist admissions set at £341 per bed day was taken from research commissioned by UK Department of Health (Brennan *et al*., 2019). The average length of specialist admissions varies (e.g. 7–14 days), however, we defined the length of specialist admissions as 10 days according to previous research conducted in the UK (Parrott *et al*, 2006).

Non-specialist admission costs for alcohol withdrawal in NHS hospitals were extracted from our previous study examining the burden of alcohol disorders on non-specialist settings (Phillips *et al*., 2019). This study identified 231,237 individuals with alcohol disorders with a mean annual total cost related to hospital admissions of £6749 per individual accounting for a mean total of 15.14 days, which equates to £446 per bed day. Those admitted with alcohol withdrawal experienced on average 3.36 admissions/year and 17.23 total bed days. Therefore 5 days (i.e. 17.23/3.36) was estimated to be the mean length of non-specialist admissions. All costs were adjusted using UK Gross Domestic Product deflator calculations (HM Treasury, 2019).

The annual number of people accessing specialist and non-specialist admissions for alcohol withdrawal from 2009/10 to 2018/19 was presented as a time series. The mean number of admissions and costs before the implementation of HSCA (Time point 1 (T1) = 2009–2014) was compared with data since the policy change (Time point 2 (T2) = 2014–2019) using the Mann–Whitney *U* test. Correlations were reported using the Spearman’s rank correlation coefficient. We utilized R language 3.5.0 and Stata 15 for these analyses.

## RESULTS

Prevalence estimates of the number of adults with alcohol dependence in potential need of specialist treatment remained static over the assessed period. However, the percentage of adults accessing specialist admissions fell from 11.3% in 2009/10 to 5.8% in 2018/19 (see Table 1).

**Table 1:** Annual reported data for specialist (NDTMS) and non-specialist admissions (HES) for alcohol withdrawal, including estimated costs

NDTMS and HES Data	2009/10	2010/11	2011/12	2012/13	2013/14	2014/15	2015/16	2016/17	2017/18	2018/19
	Pre-policy change (T1)					Post-policy change (T2)				
^a^ Number of adults in specialist alcohol treatment ^1^	88,086	88,020	86,416	87,544	91,651	89,107	85,035	80,454	75,787	74,449
Specialist Admissions (i.e. Inpatient detoxification or ‘inpatient prescribing interventions’ for alcohol) as reported by NDTMS ^2^^,^ ^3^^,^ ^4^^,^ ^5^^,^ ^6^^,^^7^										
^b^ Inpatient detoxification (*n*)	9971	9962	10,364	9935	Definition changed					
^c^ Inpatient prescribing interventions (*n*)	–	–	–	–	8546	5806	5094	4558	3881	3614
^d^ Residential/Recovery House Prescribing Interventions (n)	–	–	–	–	1280	930	918	947	839	678
^e^ Total number of Specialist Admissions (n) ^(sum b:d)^	9971	9962	10,364	9935	9826	6736	6012	5505	4720	4292
^f^ Percentage of adults in specialist alcohol treatment receiving Specialist Admission ^(e/a*100)^	11.3%	11.3%	12.0%	11.3%	10.7%	7.6%	7.1%	6.8%	6.2%	5.8%
^g^ Cost per day (uprated from £341^*^ in 2013/14 using GDP Deflator ^8^)	£318	£324	£328	£335	£341	£345	£348	£356	£363	£370
^h^ Estimated Total Cost – Specialist Admission @ 10 days ^**^ ^((e*g)*10)^	£31,708 k	£32,277 k	£33,994 k	£33,282 k	£33,507 k	£23,239 k	£20,922 k	£19,598 k	£17,134 k	£15,880 k
Non-Specialist Admissions to the NHS for Alcohol Withdrawal (F10.3) as reported by Hospital Episode Statistics (HES) ^9^										
^i^ Total number non-specialist admissions (*n*)	21,590	22,030	22,970	22,900	24,270	25,040	26,810	27,020	27,530	31,040
^j^ Cost per day (uprated from £446† in 2009/10 using GDP Deflator ^8^)	£446	£454	£460	£469	£478	£484	£488	£499	£509	£519
^k^ Estimated Total Cost: Inpatient Non-elective Care Alcohol Withdrawal @ 5 days ^†† ((i*j^)^*^ ^5)^	£48,146 k	£50,008 k	£52,831 k	£53,701 k	£58,005 k	£60,597 k	£65,416 k	£67,415 k	£70,064 k	£80,549 k
Overall Specialist and non-specialist admissions attributed to alcohol withdrawal as recorded in NDTMS and HES										
^l^ Total number of specialist and non-specialist admissions in England ^(sum e + i)^	31,561	31,992	33,334	32,835	34,096	31,776	32,822	32,525	32,250	35,332
^m^ Estimated total costs of admissions ^(sum h + k)^	£79,854 k	£82,285 k	£86,825 k	£86,983 k	£91,512 k	£83,836 k	£86,338 k	£87,013 k	£87,198 k	£96,429 k
^n^ Cost per admission ^(m/l)^	£2530	£2572	£2605	£2649	£2684	£2638	£2630	£2675	£2704	£2729
^o^ Proportion of estimated costs attributed to SPECIALIST services ^(h/m*100)^	39.7%	39.2%	39.2%	38.3%	36.6%	27.7%	24.2%	22.5%	19.7%	16.5%

Key:

^*^Taken from Brennan et al. (2019).

^**^Detoxification length estimated at 10 days reflecting usual practice (Parrott et al, 2006).

^†^Cost of inpatient bed day taken from Phillips et al. (2019).

^††^ Phillips (2016) Identified those admitted with F10.3 experienced on average 3.36 admissions/year and 17.23 total bed days suggesting each admission averaged 5 days

^1^PHE (2019) NDTMS Adults Profiles: Adult Clients in Treatment—England https://www.ndtms.net/viewit/Adult/ExecutiveSummary.aspx

^2^PHE (2014) Adult Alcohol statistics from the National Drug Treatment Monitoring System (NDTMS) 1 April 2013 to 31 March 2014.

^3^PHE (2015) Adult substance misuse statistics from the National Drug Treatment Monitoring System (NDTMS) 1 April 2014 to 31 March 2015.

^4^PHE (2016) Adult substance misuse statistics from the National Drug Treatment Monitoring System (NDTMS) 1 April 2015 to 31 March 2016.

^5^PHE (2017) Adult substance misuse statistics from the National Drug Treatment Monitoring System (NDTMS) 1 April 2016 to 31 March 2017.

^6^PHE (2018) Adult substance misuse statistics from the National Drug Treatment Monitoring System (NDTMS) 1 April 2017 to 31 March 2018.

^7^PHE (2019) Adult substance misuse statistics from the National Drug Treatment Monitoring System (NDTMS) 1 April 2018 to 31 March 2019.

^8^HM Treasury (2019) GDP Deflators at market prices, and money GDP using GDP_Deflators_Qtrly_National_Accounts_June_2019_update.xlsx

^9^PHE (2020) Local Alcohol Profiles for England (LAPE) Statistical Table for England 2020.

Before 2013/14 the mean annual number of specialist admissions remained relatively static at 10,012 admissions. However, the mean annual number of specialist admissions decreased to 5453 admissions between 2013/14 and 2018/19, a reduction of 45.5%. Overall, annual specialist admissions decreased by a mean of 4559 comparing T1 to T2, which was statistically significant (*P* = 0.01).

By contrast, there was a 43.8% increase in non-specialist admissions between 2009/10 and 2018/19, increasing by a mean of 4736 per annum comparing T1 to T2, which was also statistically significant (*P* = 0.01).

**Figure 1:**
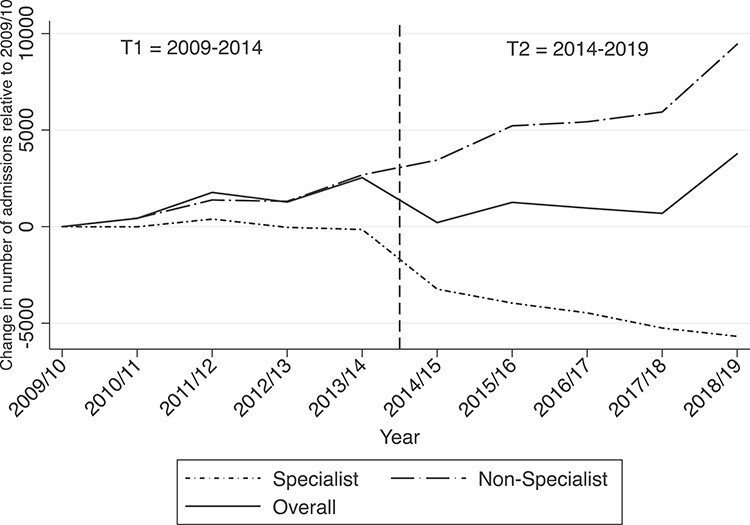
Annual changes in the number of admissions by care setting relative to 2009/10. (Reference vertical line indicates period of policy change).

Figure 1 presents changes in the number of specialist admissions and non-specialist admissions relative to a baseline in 2009/10. When considered together, the overall number of adults accessing either specialist or non-specialist admissions for alcohol withdrawal appears relatively stable, suggesting that the reductions in specialist admissions has been displaced to non-specialist admissions since 2013/14.

The annual total specialist admissions were compared with non-specialist admissions for the same years using the Spearman’s rank correlation. A negative correlation was observed (*r =* −0.93, *P* < 0.01), providing strong evidence of an association between the annual reduction in specialist admissions and the annual increase in non-specialist admissions.

The annual estimated total costs of specialist admissions reduced from £31.7m to £15.9m from 2009/10 to 2018/19. Comparing T1 and T2, these costs decreased by a mean of £13.6m per annum: a statistically significant reduction of 41.3% (*P* = 0.01). In contrast the estimated costs for non-specialist admissions increased by 67.3% between 2009 and 2019: a mean increase of £16.3m per annum comparing T1 to T2, which was statistically significant (*P* = 0.01). Despite the decrease in specialist admission costs and increases in non-specialist admission costs, the overall total cost estimates remained relative static over the reporting period with no significant difference in mean cost between T1 to T2.

The average cost per admission varied over time and between settings with a specialist admission at £3180 in 2009/10 increasing to £3700 in 2018/19, compared to non-specialist admission £2,230 and £2595 respectively. Despite the cost per day being lower for specialist admissions, the estimated length of stay was twice that of non-specialist admissions (i.e. 10 days versus 5 days). Therefore, the estimated cost of each non-specialist admission was 29.8% less than for specialist admissions. The overall cost per admission remained relatively unchanged from 2009/10 to 2018/19 and was not found to be statistically significant between T1 and T2.

## DISCUSSION

Our analysis identifies that before the introduction of the HSCA, specialist alcohol services offered approximately 10,000 specialist admissions annually. This study found from 2013/14 to 2018/19 a decline in the clinical population accessing specialist treatment and a significant rapid reduction in specialist admissions for alcohol withdrawal. The parallel increase in non-specialist admissions and the strong statistical association between these trends suggest a net substitution of care from specialist to non-specialist admissions since the implementation of the HSCA. However, the overall total number of all admissions for alcohol withdrawal interventions remained relatively static, emphasizing a continued and increasing need for inpatient treatment for alcohol withdrawal. The transfer from specialist to non-specialist admissions was also reflected in the costs for inpatient care that are met by different governmental organizations. We found a significant shift in costs for alcohol withdrawal from local authorities responsible for specialist admissions to local NHS commissioners responsible for the provision of non-specialist admissions.

It is important to acknowledge several limitations. Firstly, the definitions used to describe alcohol withdrawal admissions vary between time points and settings. It is widely acknowledged that inpatient treatment under specialist alcohol services will involve medically managed alcohol withdrawal regardless of their coding, be it ‘inpatient alcohol detoxification or inpatient prescribing interventions’. Equally, non-specialist admissions for alcohol withdrawal are based on the clinical presentation of alcohol withdrawal symptoms requiring clinical management and therefore provide a comparable data source.

Secondly, the economic analysis provides estimates based on aggregate costs and lengths of admission across both settings. We chose a more conservative period for specialist admissions based on literature that was more reflective of recent practice. The length of stay for non-specialist admissions is drawn from a national sample in 2009/10. While we feel this accurately reflects the average length of admission at this time, it is possible that variation in practice exists, skewing costs.

Thirdly, while the rapid decline in specialist admissions coincides with the service changes prompted by the HSCA, and further exacerbated by significant reductions to the public health grant to local authorities in 2015/16 (Department of Health, 2015) used to support specialist alcohol treatment, this time series analysis is unable to identify a specific causal relationship between the fall and rise in alcohol withdrawal admissions.

International literature examining alcohol treatment systems has identified that, as unmet demand for treatment decreases in one part of the system, there are increases in another part of the system (Ritter *et al*., 2019). Significant evidence of the effectiveness and cost-effectiveness of specialist alcohol treatment exists (NICE, 2011), with improved outcomes being experienced when specialist admissions are integrated with community treatment (Eastwood *et al*., 2018). Previous studies in North America and Scandinavia have identified that increases in effective specialist alcohol treatment are associated with decreases in liver morbidity (Smart *et al*., 1996), liver and all-cause mortality (Holder & Parker, 1992; Rautiainen *et al*., 2019) as well as hospital admissions (Smart & Mann, 2000). A recent governmental inquiry into the fall in the number of people entering specialist alcohol services between 2013 to 2017 concedes financial pressures increased barriers and reduced access to specialist treatment (Public Health England, 2018b). It is therefore plausible that the observed increase in non-specialist admissions may in part be influenced by the reduced ability of specialist services to provide the full recommended range of interventions including specialist admissions for alcohol withdrawal.

Equally, it might be argued that the clinical characteristics of those requiring treatment have changed over the last decade, meaning that specialist services are unable to fully meet the needs of severely ill service users experiencing alcohol withdrawal. Firstly, there has been an ageing cohort of harmful and dependent drinkers with comorbid conditions (Drummond *et al*., 2016). In addition, increasing non-specialist admissions for alcohol-related liver disease has worsened particularly in deprived areas (Williams *et al*., 2020), pointing towards greater clinical complexity. Our previous study identified that unplanned non-specialist admissions were greater in those with chronic alcohol disorders, including alcohol withdrawal, than those with an acute or no alcohol disorder (Phillips *et al*., 2019). Therefore, the shift in demand from specialist to non-specialist admissions might be best explained by reductions in funding exacerbated by the increasing complexity and clinical needs of the service users.

Despite the higher individual bed day cost, non-specialist admissions are briefer than recommended (NICE, 2011) and significantly shorter compared to specialist admissions, thereby reducing the cost of each admission. Shorter lengths of admission suggest that many will continue to experience withdrawal features on discharge, promoting potential relapse and readmission (Yedlapati and Stewart, 2018). Furthermore, socially disenfranchised groups who experience greater risk factors are less likely to engage in follow-up treatments and are more likely to be readmitted (Neighbors *et al*., 2018).

To realize the full public health benefits of alcohol treatment, there is a requirement to evaluate and develop a system of care based on population needs that ensures services are accessible, efficient and appropriately resourced (Babor *et al*., 2008; Rush & Urbanoski, 2019). Although desirable, it is unlikely that the closure of specialist inpatient units will be reversed in the short-term. We suggest the remaining specialist services should be preserved to support integrated care pathways and act as specialist training centres. The reduction in specialist admissions means that specialist care for alcohol withdrawal is being transferred to non-specialist care settings that may be less equipped to meet the current and predicted increase in alcohol-related hospital admissions (McQuire *et al*., 2019). The recent development of care pathways and hospital-based Alcohol Care Teams may promote the completion of alcohol withdrawal programmes in the community that shorten non-specialist admissions (Public Health England, 2018a; NHS England, 2019). In addition, these initiatives provide opportunities for specialist and non-specialist practitioners to develop shared competencies (Phillips *et al*., 2020), innovative service models, deliver comprehensive packages of care within the hospital and integrate effective community treatments (Moriarty, 2019; Drummond *et al*., 2019).

Previous commentators have warned of the consequences of cuts to specialist treatment services following the introduction of the HSCA (Drummond, 2017). Our analysis suggests that those in need of specialist inpatient care are likely to be disproportionately affected by changes in funding following the implementation of this policy. Service models within non-specialist care settings should evolve within an integrated model of provision to ensure the needs of service users are fully met.

## References

[ref1] Babor TF, Stenius K, Romelsjo A (2008) Alcohol and drug treatment systems in public health perspective: Mediators and moderators of population effects. Int J Methods Psychiatr Res 17:S50–9. 18543363 10.1002/mpr.249 PMC6879072

[ref2] Brennan A, Hill-McManus D, Stone T et al. (2019) Modeling the potential impact of changing access rates to specialist treatment for alcohol dependence for local authorities in England: The specialist treatment for alcohol model (STreAM). Stud Alcohol Drugs Suppl 18:96–109. 10.15288/jsads.2019.s18.96 PMC6377021 30681953

[ref3] Department of Health (2013) Localising the Public Health Responsibility Deal - a toolkit for local authorities. Department of Health. Available at: https://assets.publishing.service.gov.uk/government/uploads/system/uploads/attachment_data/file/193106/130408-RD-Toolkit-Web-version.pdf (20 October 2019, date last accessed).

[ref4] Department of Health (2015) Local authority public health allocations 2015/16: in-year savings. Department of Health. Available at: https://assets.publishing.service.gov.uk/government/uploads/system/uploads/attachment_data/file/450508/Cons_doc_HA_version.pdf (20 October 2019, date last accessed).

[ref5] Drummond C, McBride O, Fear N, et al. (2016) Chapter 10: Alcohol dependence in McManus S, Bebbington P, Jenkins R. et al (eds) Mental health and wellbeing in England: Adult Psychiatric Morbidity Survey 2014. Leeds: NHS Digital. Available at: https://files.digital.nhs.uk/pdf/r/1/adult_psychiatric_study_ch10_web.pdf (1 July 2020, date last accessed).

[ref6] Drummond C (2017) Cuts to addiction services are a false economy. BMJ 357:j2704. 28584061 10.1136/bmj.j2704

[ref7] Drummond C, Wolstenholme A, Blackwood R et al. (2019) Assertive outreach for high-need, high-cost alcohol-related frequent NHS hospital attenders: The value-based case for investment. National Institute for Health Research Collaboration for Leadership in Applied Health Research and Care South London.

[ref8] Eastwood B, Peacock A, Millar T et al. (2018) Effectiveness of inpatient withdrawal and residential rehabilitation interventions for alcohol use disorder: A national observational, cohort study in England. J Subst Abuse Treat 88:1–8. 29606222 10.1016/j.jsat.2018.02.001

[ref9] HC Deb (2019) vol. 660, col. 149. Available at: https://hansard.parliament.uk/Commons/2019-05-14/debates/D6FAC629-2148-4080-893B-DE850B227901/Health (13 November 2019, date last accessed).

[ref10] Health and Social Care Act (2012), c.7. Available at: http://www.legislation. gov.uk/ukpga/2012/7/contents/enacted (20 October 2019, date last accessed).

[ref11] Treasury HM (2019) Gross Domestic Product (GDP) deflators at market prices, and money GDP: June update using, GDP_Deflators_Qtrly_ National_Accounts_June_2019_update.xlsx. Available at: https://www.gov.uk/government/statistics/gdp-deflators-at-market-prices-and-money-gdp-june-2019-quarterly-national-accounts (13 November 2019, date last accessed).

[ref12] Holder HD, Parker RN (1992) Effect of alcoholism treatment on cirrhosis mortality: A 20-year multivariate time series analysis. Br J Addict 87:1263–74. 1327335 10.1111/j.1360-0443.1992.tb02735.x

[ref13] Jones L, Bellis MA, Dedman D et al. (2008) Alcohol-attributable fractions for England: alcohol-attributable mortality and hospital admissions. Liverpool Centre for Public Health, Liverpool John Moores University.

[ref14] McQuire C, Tilling K, Hickman M et al. (2019) Forecasting the 2021 local burden of population alcohol-related harms using Bayesian structural time–series. Addiction 114:994–1003. 30694577 10.1111/add.14568 PMC6563459

[ref15] Moriarty KJ (2019) Alcohol care teams: Where are we now. Front Gastroenterol . http://fg.bmj.com/cgi/content/full/flgastro-2019-101241. 10.1136/flgastro-2019-101241 PMC7307041 32582422

[ref16] NHS England (2019) Alcohol care team: Core Service descriptors. Available at: https://www.longtermplan.nhs.uk/wp-content/uploads/2019/11/ACT-core-service-descriptor-051119.pdf (8 February 2020, date last accessed).

[ref17] National Institute for Health and Care Excellence (2010) Alcohol-use disorders: Diagnosis and management of physical complications. Clinical guideline 100. National Institute for Health and Care Excellence.

[ref18] National Institute for Health and Care Excellence (2011) Alcohol-use disorders: Diagnosis, assessment and management of harmful drinking and alcohol dependence. Clinical guideline 115. National Institute for Health and Care Excellence. 31886968

[ref19] Neighbors CJ, Yerneni R, O'Grady MA et al. (2018) Recurrent use of inpatient withdrawal management services: Characteristics, service use, and cost among Medicaid clients. J Subst Abuse Treat 92:77–84. 30032948 10.1016/j.jsat.2018.06.013

[ref20] Parrott S, Godfrey C, Heather N et al. (2006) Cost and outcome analysis of two alcohol detoxification services. Alcohol Alcohol 41:84–91. 16272193 10.1093/alcalc/agh236

[ref21a] Phillips, T (2016) What Is the Burden of Alcohol Related Problems on Accident and Emergency Departments (AED) in England? : An Epidemiological Analysis of the Prevalence of Alcohol Use Disorders (AUDs) within the AED Setting (PhD. thesis) King's College, London. Institute of Psychiatry, Psychology & Neuroscience.

[ref21] Phillips T, Coulton S, Drummond C (2019) Burden of alcohol disorders on emergency department attendances and hospital admissions in England. Alcohol Alcohol 54:516–24. doi: 10.1093/alcalc/agz055. 33724349

[ref22] Phillips T, Porter A, Sinclair J (2020) Clinical competencies for the Care of Hospitalized Patients with alcohol use disorders. Alcohol Alcohol 55:395–400. doi: 10.1093/alcalc/agaa024. 32318727 PMC7307320

[ref23] Public Health England (2018a) Developing pathways for referring patients from secondary care to specialist alcohol treatment. Available at: https://www.gov.uk/government/publications/developing-pathways-for-alcohol-treatment/developing-pathways-for-referring-patients-from-secondary-care-to-specialist-alcohol-treatment (20 October 2019, date last accessed).

[ref24] Public Health England (2018b) PHE inquiry into the fall in numbers of people in alcohol treatment: findings available at: https://www.gov.uk/government/publications/alcohol-treatment-inquiry-summary-of-findings/phe-inquiry-into-the-fall-in-numbers-of-people-in-alcohol-treatment-findings (8 February 2020, date last accessed).

[ref25] Public Health England (2020) Local alcohol profiles for England: LAPE_Statistical_Tables_for_England_2020. Public Health England [https://fingertips.phe.org.uk/documents/LAPE_Statistical_Tables_for_England_2020.xlsx ] (17 February 2020, date last accessed).

[ref26] Rautiainen E, Ryynänen OP, Reissell E et al. (2019) Alcohol-related social and health service use patterns as predictors of death and remission in patients with AUD. J Subst Abuse Treat 96:65–74. 30466551 10.1016/j.jsat.2018.10.013

[ref27] Ritter A, Mellor R, Chalmers J et al. (2019) Key considerations in planning for substance use treatment: Estimating treatment need and demand. J Stud Alcohol Drugs Suppl 22–30. 30681945 10.15288/jsads.2019.s18.22 PMC6377022

[ref28] Roberts E, Morse R, Epstein S et al. (2019) The prevalence of wholly attributable alcohol conditions in the United Kingdom hospital system: A systematic review, meta-analysis and meta-regression. Addiction 114:1726–37. 31269539 10.1111/add.14642 PMC6771834

[ref29] Robertson R, Wenzel L, Thompson J et al. (2017) Understanding NHS Financial Pressures: How they are affecting patient care. London The King’s Fund.

[ref30] Rush B, Urbanoski K (2019) Seven Core principles of substance use treatment system design to aid in identifying strengths, gaps, and required enhancements. J Stud Alcohol Drugs Suppl 9–21. 30681944 10.15288/jsads.2019.s18.9 PMC6377009

[ref31] Smart RG, Mann RE (2000) The impact of programs for high-risk drinkers on population levels of alcohol problems. Addiction 95:37–51. 10723824 10.1046/j.1360-0443.2000.951375.x

[ref32] Smart RG, Mann RE, Lee SL (1996) Does increased spending on alcoholism treatment lead to lower cirrhosis death rates? Alcohol Alcohol 31:487–91. 8949965 10.1093/oxfordjournals.alcalc.a008183

[ref33] Williams R, Aithal G, Alexander GJ et al. (2020) Unacceptable failures: The final report of the lancet commission into liver disease in the UK. Lancet 395:226–39. 31791690 10.1016/S0140-6736(19)32908-3

[ref34] World Health Organisation (1992) The ICD-10 classification of mental and behavioural disorders: clinical descriptions and diagnostic guidelines. Geneva World Health Organisation.

[ref35] Yedlapati SH, Stewart SH (2018) Predictors of alcohol withdrawal readmissions. Alcohol Alcohol 53:448–52. 29617711 10.1093/alcalc/agy024

